# Сравнительный анализ костных осложнений при МЭН1-ассоциированном и спорадическом первичном гиперпаратиреозе

**DOI:** 10.14341/probl13385

**Published:** 2024-02-28

**Authors:** С. В. Пылина, А. К. Еремкина, А. Р. Елфимова, А. М. Горбачева, Н. Г. Мокрышева

**Affiliations:** Национальный медицинский исследовательский центр эндокринологии; Национальный медицинский исследовательский центр эндокринологии; Национальный медицинский исследовательский центр эндокринологии; Национальный медицинский исследовательский центр эндокринологии; Национальный медицинский исследовательский центр эндокринологии

**Keywords:** первичный гиперпаратиреоз, синдром множественной эндокринной неоплазии 1 типа, минеральная плотность костной ткани, трабекулярный костный индекс, менин

## Abstract

**ОБОСНОВАНИЕ:**

ОБОСНОВАНИЕ. Синдром множественной эндокринной неоплазии 1 типа (МЭН1) — редкое аутосомно-доминантное заболевание, обусловленное мутацией в гене-онкосупрессора менина (MEN1). Наиболее часто при МЭН1 поражаются околощитовидные железы с развитием первичного гиперпаратиреоза (мПГПТ). Доступные данные о суммарной частоте и выраженности костных нарушений при мПГПТ по сравнению со спорадической формой заболевания (сПГПТ) противоречивы и не позволяют сделать однозначные выводы.

**ЦЕЛЬ:**

ЦЕЛЬ. Провести сравнительный анализ клинико-лабораторных и инструментальных особенностей костных осложнений в группах мПГПТ и сПГПТ.

**МАТЕРИАЛЫ И МЕТОДЫ:**

МАТЕРИАЛЫ И МЕТОДЫ. Проведено одноцентровое ретроспективное исследование среди пациентов молодого возраста с ПГПТ в активной стадии заболевания (без паратиреоидэктомии (ПТЭ) в анамнезе). Комплексное обследование включало анализ показателей кальциево-фосфорного обмена, маркеров костного метаболизма и скрининг осложнений ПГПТ. Проводилась оценка минеральной плотности кости (МПК) методом двухэнергетической рентгеновской абсорбциометрии в поясничном отделе позвоночника, бедренной и лучевой костях, а также микроархитектоники с использованием трабекулярного костного индекса (ТКИ). Всем пациентам, включенным в исследование, выполнен генетический анализ (секвенирование гена MEN1 или секвенирование панели генов).

**РЕЗУЛЬТАТЫ:**

РЕЗУЛЬТАТЫ. В группу 1 (мПГПТ) было включено 26 пациентов, в группу 2 (сПГПТ) — 30 пациентов, сопоставимых по возрасту: медиана в группе 1 составила 34,5 года [25; 39], в группе 2 — 30,5 года [28; 36], (p=0,439, U-тест). В группе 1 была выделена подгруппа 1А (n=21), в которую вошли пациенты без гормонально-активных нейроэндокринных новообразований (НЭН) желудочно-кишечного тракта (ЖКТ) и аденогипофиза. Пациенты из групп мПГПТ и сПГПТ не различались по длительности заболевания: 1 год [0; 3] и 1 год [0; 1] соответственно (р=0,533, U-тест). Не было получено различий по основным параметрам минерального обмена, а также частоте почечной патологии. В группе мПГПТ достоверно чаще наблюдались костные нарушения в целом: 54 против 10% в сПГПТ (р=<0,001; F-тест). Выявлены статистически значимые различия как по абсолютным показателям МПК, так и по значениям Z-критерия в шейке бедренной кости и бедре суммарно, которые были ниже в группе мПГПТ. Данные различия сохраняли значимость при отдельном сопоставлении подгруппы 1А с сПГПТ.

**ЗАКЛЮЧЕНИЕ:**

ЗАКЛЮЧЕНИЕ. При мПГПТ костные осложнения заболевания встречаются чаще, чем при спорадической форме заболевания и характеризуются более низкой МПК в бедренной кости независимо от наличия/отсутствия у пациента других гормонально-активных НЭН. Уточнение роли мутации в гене MEN1 в этих процессах требует дальнейшего изучения.

## Abstract (en)

**BACKGROUND:**

BACKGROUND: Multiple endocrine neoplasia type 1 (MEN1) — is a rare syndrome with an autosomal dominant inheritance pattern caused by a mutation in the tumor suppressor gene (MEN1). Parathyroid involvement is the most common MEN1 manifestation resulting in primary hyperparathyroidism (mPHPT). Data on the prevalence and structure of bone disease in mPHPT compared to sporadic one (sPHPT) are often incomplete and contradictory.

**AIM:**

AIM: The purpose of this study was to compare the severity of bone involvement between mPHPT and sPHPT.

**MATERIALS AND METHODS:**

MATERIALS AND METHODS: A single-center retrospective study was conducted among young patients in the active phase of PHPT and without prior parathyroidectomy in anamnesis. The analysis included the main parameters of calcium-phosphorus metabolism, bone remodeling markers, as well as an assessment of disease complications. Bone mineral density (BMD) was measured using dual-energy X-ray absorptiometry (DXA) at sites of lumbar spine, femur and radius. Trabecular bone score (TBS) was applied to estimate trabecular microarchitecture. All patients included in the study underwent genetic testing.

**RESULTS:**

RESULTS: Group 1 (mPHPT) included 26 patients, and group 2 (sSHPT) included 30 age-matched patients: the median age in group 1 was 34.5 years [25; 39], in group 2 — 30.5 years [28; 36], (p=0.439, U-test). Within group 1, the subgroup 1A (n=21) was formed with patients without other hormone-produced neuroendocrine neoplasms (NEN) in the gastrointestinal tract (GI) and the anterior pituitary gland. The duration of PHPT was comparable in both groups: mPHPT — 1 year [0; 3] versus sPHPT — 1 year [0; 1], (p=0.533, U-test). There were no differences in the main parameters of calcium-phosphorus metabolism, as well as in the prevalence of kidney complications. In the mPHPT group, bone abnormalities were observed significantly more often compared to sPHPT: 54 vs 10% (p=<0.001; F-test). Statistically significant differences were revealed both in BMD and in Z-score values of the femoral neck and total hip, which were lower in the mPHPT group. These differences remained significant when comparing subgroup 1A with sPHPT.

**CONCLUSION:**

CONCLUSION: MEN1-associated PHPT may be accompanied by a more severe decrease in BMD in the femoral neck and total hip compared to sPHPT regardless of the other hormone-producing NEN. Clarifying the role of mutation in the MEN1 gene in these processes requires further study.

## ОБОСНОВАНИЕ

Синдром множественных эндокринных неоплазий 1 типа (МЭН1) — редкое аутосомно-доминантное заболевание, характеризующееся развитием опухолей эндокринных и неэндокринных тканей вследствие гетерозиготной мутации в гене онкосупрессорного белка менина (MEN1) [1]. Чаще всего при МЭН1 поражаются околощитовидные железы (ОЩЖ), нейроэндокринные клетки желудочно-кишечного тракта (ЖКТ) и передней доли гипофиза. Множественные, синхронные или метахронные новообразования ОЩЖ, приводящие к первичному гиперпаратиреозу (ПГПТ), развиваются почти у 100% пациентов с мутацией в гене MEN1 к возрасту 55 лет; опухоли ОЩЖ являются первым клиническим проявлением синдрома примерно в 90% случаев заболевания [2].

К основным отличиям ПГПТ, обусловленного синдромом МЭН1 (мПГПТ), от спорадической формы заболевания (сПГПТ) относят: более молодой возраст манифестации (25 против 55 лет), отсутствие гендерных различий [3], множественное поражение ОЩЖ, гистологически чаще являющееся гиперплазией, а не аденомой [4]. Кроме того, в ряде работ отмечалось более мягкое течение ПГПТ, оцениваемое по сывороточным концентрациям паратиреоидного гормона (ПТГ) и кальция у пациентов с мПГПТ [5–7]. Данные по ПГПТ-ассоциированным осложнениям при МЭН1 остаются неоднозначными. Lourenco DM и соавт. описали высокую распространенность раннего дебюта нефролитиаза у пациентов с МЭН1 (до 86,2% у лиц моложе 30 лет) [8], в другой работе частота нефролитиаза при МЭН1 была сопоставима с сПГПТ [9]. В ряде публикаций сообщалось о более тяжелых костных нарушениях при мПГПТ по сравнению со спорадической формой заболевания. Предположительно, данные различия могут быть связаны с более ранними потерями минеральной плотности кости (МПК) в связи в свзяи с дебютом заболевания в молодом возрасте и более длительной экспозицией высоких концентраций ПТГ, что приводит к выраженным потерям плотности кортикальной кости. Также рассматривается роль мутации в гене менина в патогенезе костных нарушений. Изучение особенностей костного метаболизма при мПГПТ является важным шагом на пути к персонализации терапевтических подходов в лечении, в частности при мягких формах, рецидиве заболевания или отказе от хирургического вмешательства. Оценка показателей МПК при рентгеновской денситометрии (как наиболее часто используемого метода диагностики снижения МПК ниже ожидаемых значений) у молодых пациентов с мПГПТ может быть сопряжена с рядом трудностей. Чем моложе пациент, тем сложнее интерпретировать результаты исследования по сравнению с аналогичными измерениями у взрослых. Результаты исследования в популяции детей и подростков могут значимо меняться с течением времени, что будет отражать накопление минеральной плотности по мере увеличения длины и ширины костей. Прирост МПК в норме отмечается на втором и даже третьем десятилетии жизни, когда достигается пик костной массы, потому так важна оценка результатов рентгеновской денситометрии именно в динамике [10]. Еще одна проблема заключается в том, что данные по МПК и риску переломов у пациентов с мПГПТ часто неполные [11] и отражают изменения в поясничном отделе позвоночника и проксимальном отделе бедренной кости. Анализ МПК лучевой кости и оценка трабекулярного костного индекса (ТКИ) представлены лишь в нескольких пилотных исследованиях [8][12]. Кроме того, негативное влияние на МПК, помимо раннего дебюта мПГПТ, влияющего на набор пика костной массы [13], могут оказывать и другие компоненты синдрома (гормонально-активные опухоли ЖКТ, передней доли гипофиза, надпочечников), а также ассоциированные с ними методы хирургического и медикаментозного лечения [14]. Так, недавно Altieri и соавт. [15] продемонстрировали большую частоту снижения МПК у пациентов с МЭН1 и нейроэндокринными новообразованиями (НЭН) ЖКТ, что прежде всего было связано с алиментарной недостаточностью по причине гиперсекреции гастроинтестинальных гормонов, медикаментозной терапии аналогами соматостатина и/или химиотерапии, а также синдрома мальабсорбции после обширных хирургических вмешательств на двенадцатиперстной кишке и поджелудочной железе.

Кроме того, ограниченность охвата генетическим скринингом пациентов молодого возраста с ПГПТ не позволяет вовремя идентифицировать лиц с мПГПТ, которые на момент манифестации заболевания не соответствуют клиническим и семейным критериям диагноза МЭН1 [3][16]. Сочетание вышеуказанных факторов создает предпосылки для гетерогенности и малочисленности оцениваемой выборки ввиду орфанности данного заболевания.

## ЦЕЛЬ ИССЛЕДОВАНИЯ

Исследовать клинико-лабораторные и инструментальные особенности костных осложнений мПГПТ по сравнению со спорадической формой заболевания.

## МАТЕРИАЛЫ И МЕТОДЫ

## Место и время проведения исследования

Исследование было проведено на базе ГНЦ РФ ФГБУ «НМИЦ эндокринологии» Минздрава России (далее — Центр). В исследование были включены пациенты, находившиеся на стационарном лечении в Центре с 27.05.2011 по 05.07.2023 гг. и соответствовавшие критериям включения в исследование.

## Изучаемые популяции

Целевая популяция — пациенты с верифицированной мутацией в гене MEN1 и подтвержденным ПГПТ в активной фазе заболевания. Группа сравнения — лица с ПГПТ в активной фазе заболевания без мутации в гене MEN1, сопоставимые по возрасту. Внутри целевой популяции выделены подгруппы: 1А — наличие ПГПТ с исключением других гормонально-активных опухолей, 1Б — наличие ПГПТ в сочетании с другими гормонально-активными опухолями.

## Способ формирования выборки из изучаемой популяции

В данном исследовании использовался метод подбора пар.

## Дизайн исследования

Проведено одноцентровое ретроспективное неконтролируемое исследование. Итоговая выборка состояла из 56 пациентов (рис. 1).

**Figure:**
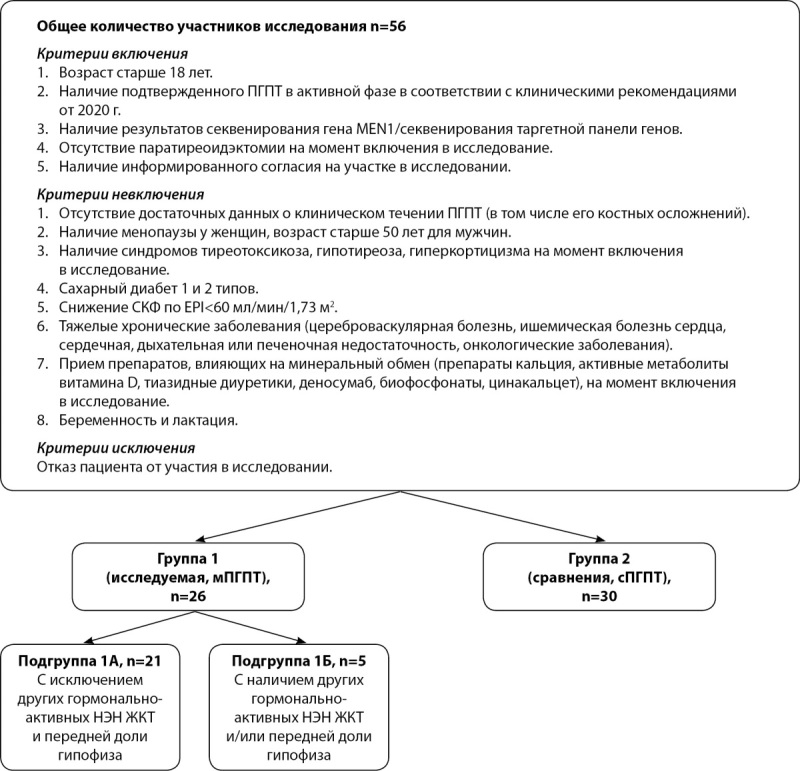
Рисунок 1. Дизайн исследования.мПГПТ — МЭН1-ассоциированный первичный гиперпаратиреоз с наличием мутации в гене MEN1; cПГПТ — спорадическая форма первичного гиперпаратиреоза с отсутствием мутации в гене MEN1.

## Методы

C использованием автоматического биохимического анализатора ARCHITECT c8000 (Abbott, США) получены значения следующих биохимических показателей сыворотки крови: кальция общего (референсный интервал (РИ) 2,15–2,55 ммоль/л), альбумина (РИ 34–48 г/л для женщин, РИ 35–50 г/л для мужчин), фосфора (РИ 0,74–1,52 ммоль/л), креатинина (РИ 50–98 мкмоль/л для женщин, 63–110 мкмоль/л для мужчин), щелочной фосфатазы (РИ 40–150 Ед/л), а также кальция суточной мочи (РИ 2,5-8,0 ммоль/л). Определение интактного ПТГ (РИ 15–65 пг/мл), остеокальцина (РИ для женщин 11–43 нг/мл; для мужчин 14–42 нг/мл) и С-концевого телопептида коллагена 1 типа (РИ 0,3–1,1 нг/мл для женщин, РИ 0,1–0,85 нг/мл для мужчин) выполнено на электрохемилюминесцентном анализаторе COBAS 6000 (Roche). Перерасчет концентрации кальция крови с поправкой на уровень альбумина осуществлялся по формуле: альбумин-скорректированный кальций (ммоль/л) = измеренный общий кальций сыворотки крови (ммоль/л) + 0,02 х (40 — измеренный уровень альбумина г/л). Расчетная скорость клубочковой фильтрации (рСКФ) была определена с использованием формулы CKD-EPI 2009.

Для топической диагностики образований ОЩЖ при наличии показаний к хирургическому лечению ПГПТ использовались следующие методы: ультразвуковое исследование околощитовидных желез на аппарате Aplio 500 линейный датчик 14L5, 18L7 и Voluson E8, сцинтиграфия с Tc-99m — Технетрилом (MIBI) на системе ОФЭКТ/КТ Discovery NM/CT 670.

Для скрининга осложнений ПГПТ применялось ультразвуковое исследование почек на аппаратах Voluson E8 (датчиками RAB6-D, С1-5, GE Healthcare), Aplio 500 (датчиком 6С1,Toshiba); измерение МПК методом двухэнергетической рентгеновской абсорбциометрии (DEXA) и ТКИ на аппаратах Lunar iDXA и Hologic Discovery А (GE Healthcare) в поясничном отделе позвоночника (LS), шейке бедренной кости (FN), бедренной кости суммарно (TH), лучевой кости суммарно (RT) и дистальной трети лучевой кости (R33%). Диагноз фиброзно-кистозного остеита устанавливался на основе результатов мультиспиральной компьютерной томографии (МСКТ) (Revolution CT, Optima CT) или рентгенографическом исследовании (система рентгенодиагностическая с дистанционным управлением Optima RF420).

Генетическое исследование выполнялось двумя способами: 7 пациентам из группы 1 (27%) и 6 пациентам из группы 2 (20%) было проведено массовое параллельное секвенирование панели генов (AIP, AP2S1, CASR, CDC73, CDKN1A, CDKN1B, CDKN1C, CDKN2A, CDKN2C, CDKN2D, DICER1, FAM111A, GATA3, GCM2, GNA11, GNAS, MEN1, POU1F1, PRKAR1A, PRKCA, PTEN, PTTG2, SDHA, SDHB, SDHC, SDHD, TBCE) на платформе Illumina методом парно-концевого чтения, остальным пациентам проводилось полное секвенирование гена MEN1 по методу Сэнгера на приборе Genetic Analyzer 3500 (Thermo Scientific). Пробоподготовка и выделение ДНК из лимфоцитов периферической крови осуществлялось с использованием автоматической станции Auto-Pure 96 (Allsheng) и набора NucleoSpin96 Blood. Количественная и качественная оценка ДНК выполнялась на спектрофотометре BioSpectrometer (Eppendorf).

Оценка соответствия пациентов критериям включения/невключения осуществлялась на основании данных анамнеза, а также представленной медицинской документации (результаты биохимических и гормональных анализов крови, инструментальных методов исследования).

В дополнение к вышеописанному пациентам проводилась оценка уровня ТТГ (РИ 0,25–3,5 мМЕ/л, ARCHITECT I2000SR), глюкозы крови натощак (ГЛК, РИ 3,1–6,1 ммоль/л, ARCHITECT c8000, Abbott). При выявлении отклонений от РИ выполнялся пероральный глюкозотолерантный тест (ПГТТ) согласно стандартному алгоритму. При выявлении инциденталомы надпочечника или клинических признаков гиперкортицизма выполнялся ночной подавляющий тест с 1 мг дексаметазона (включение в исследование при подавлении сывороточного кортизола менее 50 нмоль/л).

Топическая диагностика НЭН брюшной полости и забрюшинного пространства осуществлялась с применением МСКТ (Revolution CT, Optima CT), визуализация опухолей гипофиза проводилась в ходе МРТ головного мозга с внутривенным контрастированием (GE SIGNA Pioneer). В рамках биохимического и рентгенологического скрининга компонентов синдрома МЭН1 анализировались следующие показатели: ИФР1 (РИ: 51–271 нг/мл для женщин; 62–230 нг/мл для мужчин; LIAISON) — при наличии отклонений ИФР1 от РИ проводился анализ уровня СТГ (РИ 0,06–6,9 нг/мл, для женщин, 0,02–1,23 нг/мл, для мужчин; LIAISON) с применением ОГТТ (оценка уровня СТГ после перорального приема 75 г глюкозы через каждые 30 минут в течение двух часов); пролактин (РИ 102–496 мЕд/л для женщин; 86–324 мЕд/л для мужчин) — при наличии клинических проявлений гиперпролактинемии, нарушении менструального цикла и/или выявлении инциденталомы гипофиза; при наличии НЭН ЖКТ, по данным КТ органов брюшной полости, — хромогранин А (РИ<3 нмоль/л; ELISA, DiaSourse Diagnostics), гастрин (РИ 13–115 пг/мл, анализ на базе ЦМД — Центра молекулярной диагностики, методом ИХЛА). При наличии специфических жалоб или отклонений уровня гастрина от РИ выполнялась эзофагогастродуоденоскопия на аппарате Olympus GIF-XP. При необходимости проводилась биопсия слизистой желудка с последующей оценкой морфологического варианта опухоли.

## Статистический анализ

Статистический анализ исследуемых групп выполнен с помощью пакета прикладных программ Statistica 13 (Tibco, США). Описательная статистика количественных переменных представлена медианами, первым и третьим квартилем Me [Q1, Q3], категориальных — абсолютными и относительными частотами (n (%)).

Сравнительный анализ количественных признаков двух независимых групп проведен с использованием критерия Манна-Уитни (U-тест). Сравнительный анализ двух независимых групп по категориальным признакам проведен с помощью точного двустороннего критерия Фишера. Корреляционный анализ выполнен с помощью метода ранговой корреляции Спирмена. Уровень значимости (p-value) при проверке статистических гипотез принимался равным 0,05. Для коррекции проблемы множественных сравнений применялся метод Холма (р0-value). Значения p-value меньше 0,05, но выше уровня статистической значимости интерпретировались как статистическая тенденция.

## Этическая экспертиза

Протокол исследования рассмотрен и одобрен на заседании локального этического комитета ФГБУ «НМИЦ эндокринологии» Минздрава России от 17.01.2018 (протокол №1). Протокол исследования и информированного согласия соответствует этическим принципам, принятым в Хельсинской декларации на 18-й Генеральной Ассамблее ВМА, Хельсинки, Финляндия, июнь 1964 г., «Этические принципы проведения медицинских исследований с участием человека в качестве субъекта» и измененным на 64-й Генеральной Ассамблее ВМА, Форталеза, Бразилия, октябрь 2013 г.

## РЕЗУЛЬТАТЫ

## Характеристика групп мПГПТ/сПГПТ

В группу 1 были включены 26 пациентов, в группу 2 — 30 пациентов. Обе группы были сопоставимы по возрасту: 34,5 года [ 25; 39] в группе 1, 30,5 лет [ 28; 36] в группе 2, (p=0,439, U-тест), а также по длительности течения ПГПТ: 1,0 год [ 0; 3] и 1,0 год [ 0; 1] соответственно (р=0,533, U-тест). В обеих группах преобладали женщины, однако соотношение мужчин и женщин было ниже в группе сПГПТ (1:3 против 1:7,5 р=0,719, F-тест). ПГПТ был первым проявлением синдрома МЭН1 у 20 из 26 (77%) пациентов (мПГПТ подгр. А+Б). В подгруппе 1Б (n=5), помимо опухолей ОЩЖ, были выявлены следующие гормонально активные образования: пролактинома (n=4, 80%); инсулинома (n=2, 40%), гастринома (n=1, 20%). В группе 2 у двоих пациентов (7%) было обнаружено гормонально неактивное образование гипофиза.

Группы мПГПТ и сПГПТ не различались по основным параметрам кальций-фосфорного обмена, а также по сывороточным концентрациям маркеров костного ремоделирования (табл. 1). В обеих группах отмечалась высокая частота нефролитиаза: в группе 1 — 38%, в группе 2 — 53% (р=0,295; F-тест). Частота костных нарушений была достоверно выше среди пациентов с мПГПТ (54 против 10%, р=<0,001; F-тест).

**Table:** Таблица 1. Сравнительный анализ обеих групп по показателям минерального обмена и результатам двухэнергетической рентгеновской абсорбциометрии (DXA) Примечание: данные представлены медианами, первым и третьим квартилями: [Q1; Q3]. При сравнении показателей использовался критерий Манна-Уитни (U-тест). Статистически значимые различия выделены жирным шрифтом. *Сравнение показателей с использованием точного двустороннего критерия Фишера (p-value; F-тест). Коррекция проблемы множественных сравнений — метод Холма (p0-value). Суммарная частота костных осложнений — снижение МПК по Z-критерию ниже (-2SD). LS lumbar spine (поясничный отдел позвоночника), FN femur neck (шейка бедренной кости), TH total hip (бедренная кость суммарно), RT radius total (лучевая кость суммарно), R33% radius 33% (дистальная треть лучевой кости), ТКИ (трабекулярный костный индекс).

Параметр	мПГПТподгр. А+Б	мПГПТподгр. А	сПГПТ	МЭН1 (+) гр. А+B vs cПГПТ	МЭН1 (+) гр. А vs cПГПТ
N	Me [ Q1; Q3]	N	Me [ Q1; Q3]	N	Me [ Q1; Q3]	P-value	P0-value	P-value	P0-value
ПТГ, пг/мл	26	131,6[ 94,3; 166,8]	21	116,5 [ 91,8; 158,0]	30	121,3 [ 106,7; 156,0]	0,967	0,025	0,509	0,010
Са скорр. ммоль/л	23	2,69[ 2,62; 2,82]	18	2,67 [ 2,61; 2,87]	29	2,65 [ 2,56; 2,76]	0,484	0,008	0,661	0,013
Фосфор, ммоль/л	21	0,79[ 0,73; 0,92]	18	0,795 [ 0,73; 0,98]	30	0,80 [ 0,72; 0,89]	0,848	0,017	0,733	0,025
СКФ (EPI) мл/мин/1,73 м²	24	110[ 97; 116]	19	110 [ 95; 117]	29	101 [ 93; 110]	0,348	0,006	0,342	0,007
Са суточной мочи, ммоль/л	22	8,840[ 6,783; 10,682]	17	8,940 [ 7,500; 10,682]	30	8,156 [ 5,910; 10,192]	0,529	0,010	0,425	0,008
Остеокальцин, нг/мл	18	55,89[ 32,89; 71,79]	15	41,25 [ 32,28; 71,79]	27	46,73 [ 34,46; 61,16]	0,635	0,013	0,958	0,050
С-концевой телопептид коллагена 1-типа, нг/мл	17	1,02[ 0,75; 1,28]	14	0,97 [ 0,60; 1,26]	27	0,84 [ 0,60; 1,2]	0,426	0,007	0,731	0,017
Щелочная фосфатаза Ед/л	19	77[ 69; 98]	16	76 [ 69; 95]	28	72 [ 50; 83]	0,055	0,004	0,102	0,005
Нефролитиаз	26	10 (38%)	-	-	30	16 (53%)	0,295*	0,006	-	-
Компрессионные переломы позвоночника	26	0 (0%)	-	-	30	1 (3%)	1,000*	0,050	-	-
Внепозвоночные переломы	26	2 (8%)	-	-	30	0 (0%)	0,211*	0,006	-	-
Суммарная частота костных нарушений	26	14 (54%)	-	-	30	3 (10%)	<0,001*	0,002	-	-
МПК г/см² LS	24	1,016[ 0,927; 1,109]	19	1,016 [ 0,931; 1,110]	27	1,140 [ 1,069; 1,217]	0,003	0,003	0,008	0,004
Z-критерий LS	24	-1,5[ -1,1; -0,8]	19	-1,4 [ -1,9; -0,3]	27	-0,5 [ -1,1; 0,0]	0,004	0,003	0,020	0,004
МПК г/см² FN	25	0,799[ 0,705; 0,948]	20	0,804 [ 0,721; 0,951]	27	0,968 [ 0,900; 1,071]	<0,001	0,002	0,001	0,003
Z критерий FN	25	-1,5[ -1,9; -1,0]	20	-1,4 [ -1,9; -0,8]	27	-0,4 [ -0,8; 0,2]	<0,001	0,002	<0,001	0,003
МПК г/см² TH	25	0,830[ 0,721; 0,922]	20	0,827 [ 0,725; 0,956]	27	0,975 [ 0,904; 1,117]	0,001	0,003	0,001	0,003
Z критерий TH	25	-1,1[ -1,9; -0,4]	20	-1,15 [ -1,85; -0,45]	27	-0,4 [ -0,8; 0,7]	0,002	0,003	0,001	0,003
МПК г/см² RT	24	0,579[ 0,515; 0,652]	19	0,582 [ 0,519; 0,674]	29	0,650 [ 0,596; 0,693]	0,039	0,004	0,140	0,006
Z-критерий RT	23	-1,3[ -2,7; -0,2]	18	-1,1 [ -2,6; 0,0]	28	-0,6 [ -1,3; 0,2]	0,040	0,004	0,149	0,006
МПК г/см² R33%	24	0,740[ 0,677; 0,840]	19	0,737 [ 0,679; 0,857]	29	0,828 [ 0,776; 0,869]	0,017	0,003	0,039	0,004
Z-критерий R33%	23	-1,5[ -2,3; -0,5]	18	-1,6 [ -2,3; -0,5]	28	-0,5 [ -1,1; -0,1]	0,003	0,003	0,007	0,003
ТКИ (РИ>1,310)	9	1,386[ 1,318; 1,448]	6	1,356 [ 1,318; 1,448]	16	1,487 [ 1,340; 1,513]	0,095	0,005	0,060	0,005

Фиброзно-кистозный остеит (ФКО) был выявлен у двух (8%) пациентов из группы мПГПТ. У пациентки 44 лет с максимальным снижением МПК в лучевой кости до -4,4 SD по Z-критерию отмечались множественные «бурые» опухоли костей таза, ребер и большеберцовых костей; у молодого пациента 22 лет с максимальным снижением МПК в FN до -3,0 SD по Z-критерию «бурые» опухоли локализовались в костях правой голени, левой плечевой кости, левой ключице и левой бедренной кости. В группе сПГПТ ни у одного из включенных пациентов признаков ФКО не наблюдалось. При этом частота низкоэнергетических переломов (внепозвоночных и компрессионных) в обеих группах была сопоставима (табл. 1).

При более детальном анализе состояния костной ткани в различных отделах нами были выявлены статистически значимые различия как по абсолютным показателям МПК, так и по значениям Z-критерия в шейке бедренной кости и бедре суммарно, которые были ниже в группе мПГПТ. Более того, данные различия сохраняли свою значимость при отдельном сопоставлении подгруппы 1А со спорадической формой заболевания. Расхождения между группами в абсолютных значениях МПК и Z-критерия в поясничном отделе позвоночника и лучевой кости находились на уровне статистических тенденций. Показатели ТКИ в обеих группах были сопоставимы (табл. 1).

Для выявления потенциальной взаимосвязи между тяжестью костных проявлений и отклонениями параметров фосфорно-кальциевого обмена вследствие ПГПТ нами был проведен корреляционный анализ (табл. 2). Статистически значимая отрицательная корреляция установлена между уровнем остеокальцина и МПК лучевой кости суммарно только в группе сПГПТ. Остальные паттерны взаимосвязей в обеих группах были различны и находились на уровне статистических тенденций.

**Table:** Таблица 2. Корреляционный анализ. Взаимосвязи показателей кальциево-фосфорного обмена с МПК и ТКИ внутри группы 1 и 2 Примечание: корреляционный анализ (р, метод Спирмена). Коррекция проблемы множественных сравнений — метод Холма (p0-value). Жирным цветом выделены статистически значимые взаимосвязи. Остальные параметры находятся на уровне статистической тенденции. LS lumbar spine (поясничный отдел позвоночника), FN femur neck (шейка бедренной кости), TH total hip (бедренная кость суммарно), RT radius total (лучевая кость суммарно), R33% radius 33% (дистальная треть лучевой кости), ТКИ (трабекулярный костный индекс).

Группа 1 (мПГПТ А+Б)	Группа 2 (сПГПТ)
Параметры	N	p, метод Спирмена	р0	r	N	p, метод Спирмена	р0	r
МПК LS г/см²	Остеокальцин, нг/мл	17	0,020	0,002	-0,56	-	-	-	-
МПК FN, г/см²	Остеокальцин, нг/мл	18	0,049	0,002	-0,47	-	-	-	-
МПК TH, г/см²	Щелочная фосфатаза, Ед/л	18	0,016	0,002	-0,56	-	-	-	-
МПК RT, г/см²	ПТГ, пг/мл	-	-	-	-	29	0,048	0,002	-0,37
Остеокальцин, нг/мл	-	-	-	-	26	<0,001	0,002	-0,66
МПК R33%, г/см²	Кальций суточной мочи, ммоль/сут	-	-	-	-	29	0,047	0,002	-0,37
Остеокальцин, нг/мл	18	0,046	0,002	-0,48	26	0,011	0,002	-0,49
ТКИ	Кальций суточной мочи, ммоль/сут	-	-	-	-	16	0,031	0,002	0,54
С-концевой телопептид коллагена 1-типа, нг/мл	6	0,050	0,002	0,81	-	-	-	-

## ОБСУЖДЕНИЕ

В настоящей работе представлены результаты первого пилотного российского исследования по сравнению костно-метаболических нарушений у пациентов молодого возраста с МЭН1-ассоциированной и спорадической формой ПГПТ в активной фазе заболевания. От аналогичных работ настоящее исследование отличается молекулярно-генетическим подтверждением/исключением диагноза МЭН1, а также введенными при формировании выборок жесткими возрастными ограничениями [6–7][9][11–12][14][17–18]. В настоящем исследовании различий между группами по показателям кальциево-фосфорного обмена и концентрациям сывороточных маркеров костного метаболизма выявлено не было. При этом в исследовании Eller-Vainicher C. и соавт. концентрации ПТГ (113,8±69,5 против 173,7±135 пг/мл, p=0,001) и фосфора (2,38±0,52 против 2,56±0,45 мг/дл; p=0,003) были значимо ниже в группе мПГПТ по сравнению с лицами со спорадической формой заболевания [6]. Аналогичный результат в отношении ПТГ был продемонстрирован Kong J. и соавт. (4,0 верхних границ нормы локальной лаборатории при мПГПТ против 9,8 верхних границ при сПГПТ; р<0,001), при этом уровень фосфора был ниже в группе сПГПТ (0,73 против 0,84 ммоль/л, p<0,05) [21]. В работе Lamas C. и соавт. преобладали мягкие или бессимптомные формы. Уровни ПТГ и кальция не были связаны со степенью снижения МПК. Однако значения ПТГ при мПГПТ были выше при наличии нефролитиаза Me [Q1; Q3] 15,3 [ 11,8; 19,9] против 8,9 [ 6,8; 12,2] пмоль/л, р=0,001 [7].

Отличие результатов нашей работы от ранее опубликованных исследований может быть обусловлено различной длительностью ПГПТ, которая в нашем исследовании составила всего 1 год и была сопоставима в обеих группах (р=0,533, U-тест). С другой стороны, по данным Всероссийского регистра, пациенты с сПГПТ и мПГПТ также не различаются по параметрам минерального обмена, плюс наблюдается тенденция к более выраженной гиперкальциемии у пациентов с МЭН1 [19]. Нельзя исключить, что это может быть клинической особенностью российской популяции пациентов с мПГПТ.

В нашем исследовании группы не различались по показателям СКФ, суточной кальциурии; частота структурных нарушений со стороны почек была также сопоставима в обеих группах. Lamers C. и соавт. и Eller-Vainicher C. и соавт. не выявили разницы в распространенности поражения почек в целом между сПГПТ и мПГПТ [6][20]. Однако в работе Kong J. и соавт. симптомный нефролитиаз все же чаще отмечался среди пациентов с МЭН1 синдромом (60 против 40,2%) [21].

В нашем исследовании частота костных нарушений в группе мПГПТ была значительно выше, чем в группе сПГПТ (54 против 10%) (р=<0,001), что согласуется с данными Song А. и соавт. [12], где эти показатели составили 22,4 против 10,8% соответственно (р=0,002). Coutinho L. и соавт. также выявили высокую частоту костной патологии [5]: снижение МПК ниже ожидаемых по возрасту значений (<-2,0 SD по Z-критерию) при мПГПТ до проведения ПТЭ выявлялось во всех исследуемых областях: в 50% случаев в дистальной трети лучевой кости, в 43,7% случаев в поясничном отделе позвоночника и лучевой кости суммарно, в 25% случаев в шейке бедренной кости и в 18,8% случаев в бедренной кости суммарно.

В соответствии с классическими представлениями о патофизиологии костной ткани, высокий уровень ПТГ оказывает негативное влияние прежде всего на кости с преобладанием кортикального слоя (шейка бедренной кости и лучевая кость), и значимо меньшее — на трабекулярную костную ткань (например, поясничный отдел позвоночника) [21]. Однако, в отличие от результатов DEXA и гистоморфометрических исследований, оценка ТКИ и периферическая количественная компьютерная томография высокого разрешения (HRpQCT) свидетельствуют о негативных изменениях во всех исследуемых отделах [22].

Так, в исследовании Santos LMD. и соавт. [23] было продемонстрировано, что ПГПТ также приводит к нарушению микроархитектоники костной ткани (ТКИ при ПГПТ=1,233 против ТКИ в группе контроля=1,280; p=0,044), а по результатам ROC анализа ТКИ<1,187 был ассоциирован с высоким риском позвоночных переломов. Схожие данные были получены Song А. и соавт. [11], где ТКИ при мПГПТ составил 1,22±0,14 против 1,29±0,11 при сПГПТ, р=<0,001.

В исследованиях Eller-Vainicher C. и соавт. и Mathew U. и соавт. более низкие показатели МПК при мПГПТ по сравнению с сПГПТ были выявлены не только в шейке бедренной кости, но и в поясничном отделе позвоночника [6][18], однако результаты были получены только по суррогатным показателям (Z и T критериям), а не по абсолютным значениям МПК. В исследовании Marini F. и соавт. [10] различий в МПК между группами не было выявлено, что потенциально могло быть связано с возраст-ассоциированным влиянием на костную ткань, поскольку группы не были сопоставимы по данному показателю (мПГПТ 38,9 лет±16,1 против сПГПТ 64,3 лет ±14,1 p=<0,01).

Нами было выявлено статистически значимое снижение МПК в шейке бедренной кости и бедре суммарно (табл. 1), как по абсолютным значениям, так и по Z-критерию. Более того, указанные различия выявлены не только в основной группе мПГПТ, но и в подгруппе 1А. Наличие статистически значимых изменений именно в бедренной кости в дальнейшем (при подтверждении данных на больших выборках) может быть учтено при выборе антирезорбтивных средств с наибольшим влиянием именно на этот отдел. Намечены тенденции в показателях МПК в поясничном отделе позвоночника при сПГПТ и мПГПТ, однако различия не достигли статистической значимости после применения поправки на множественные сравнения. Также мы не выявили статистических различий в значениях по МПК в поясничном отделе позвоночника и ТКИ (ТКИ мПГПТ 1,386 против ТКИ сПГПТ 1,487; р=0,095). Таким образом, мы не подтвердили более значимые нарушения микроархитектоники и более тяжелую патологию трабекулярной костной ткани при МЭН1, при этом МЭН1-синдром был ассоциирован с более тяжелым поражением кортикальной ткани бедренной кости. Несмотря на то что при ПГПТ, наряду с классическими отделами [24] при рентгеноденситометрическом исследовании, рекомендовано оценивать МПК в лучевой кости [25], последняя крайне редко используется в исследованиях. Так Lourenço DM. и соавт. при изучении нескольких семей с мПГПТ продемонстрировали, что у лиц младше 50 лет деминерализация дистальной трети лучевой кости характеризуется выраженным снижением МПК по Z-критерию и встречается в 40% случаев, в отличие от шейки бедренной кости (25%) и поясничного отдела позвоночника (23,8%), что согласуется с нашими данными в группе мПГПТ: лучевая кость суммарно (33%), дистальная треть лучевой кости (29%), поясничный отдел позвоночника (24%), шейка бедренной кости (16%).

В нашем исследовании при сравнении мПГПТ/сПГПТ показатели МПК в лучевой кости и поясничном отделе позвоночника не достигли статистической значимости, однако сохранялись на уровне статистической тенденции. С учетом реализованного дизайна (исходно сопоставимых значений возраста и длительности заболевания) это может быть связано с недостаточной мощностью исследования, а статистическая значимость может быть достигнута при увеличении объема выборок.

В исследовании Song A. и соавт. значимых различий по частоте костных осложнений (патологические переломы, фиброзный остеит, остеомаляция и субпериостальная резорбция) в подгруппах мПГПТ/сПГПТ, сопоставимых по возрасту, выявлено не было; изменения встречались в 11,6–12,7% случаев [12]. Аналогичные данные были получены Mathew EU и соавт. (24,9% против 13,0%, p=0,2) [18], что согласуется с нашими результатами.

Выявленные различия по частоте костных нарушений между мПГПТ/сПГПТ, особенно при выделении подгруппы 1А, при отсутствии статистически значимых различий по маркерам костного метаболизма, могут свидетельствовать о наличии особых патофизиологических механизмов, обуславливающих природу костных нарушений при синдроме МЭН1. Не исключено непосредственное влияние мутации в гене MEN1 на остеогенез. Результаты экспериментальных работ свидетельствуют о том, что менин является не только белком-онкосупрессором, но и поддерживает минерализацию костного матрикса на ранних этапах дифференцировки мезенхимальных клеток в остеобласты [26]. Среди потенциальных механизмов рассматривается участие менина в сигнальном пути костного морфогенетического белка (BMP2); взаимодействие с транскрипционными факторами JunD и RUX2, регулирующими дифференцировку клеток остеобластического ряда на стадии прогениторов [27]. Liu P. и соавт. с использованием животных моделей показали, что дефицит менина приводит к преобладанию остеокластогенеза над остеобластогенезом вследствие увеличения экспрессии интерферон-гамма индуцибельного протеина 10 (CXCL10), который увеличивает активность лиганд рецептора-активатора ядерного фактора каппа-B (RANKL) [28].

## Ограничения исследования

Основными ограничениями нашего исследования являются относительно небольшой объем выборок, ретроспективный дизайн, что прежде всего связано с редкостью заболевания. В рамках генетического анализа не всем пациентам было проведено секвенирование панели генов, ассоциированными с редкими наследственными формами ПГПТ. В ряде случаев было использовано только секвенирование гена MEN1. В представленном исследовании не проводился анализ уровня 25(ОН)D, так как данный показатель не входит в стандарт обследования больных с ПГПТ.

## Направления дальнейших исследований

В качестве направления для дальнейшего исследования нами планируется оценка показателей кальций-фосфорного обмена, а также МПК через 1 год после хирургического лечения ПГПТ в обеих группах.

## ЗАКЛЮЧЕНИЕ

В результате проведенного пилотного исследования впервые в российской популяции было наглядно продемонстрировано более частое поражение костной ткани при ПГПТ, ассоциированном с МЭН1 синдромом у молодых пациентов. Наиболее значимые изменения по сравнению со спорадической формой заболевания были отмечены для бедренной кости. Полученные результаты обосновывают необходимость дальнейшего исследования обозначенной проблемы как для уточнения патогенеза поражения костной ткани при мутации в гене MEN1, так и поиска новых мишеней для таргетной терапии, что в будущем позволит персонализировать подходы в лечении для данной категории пациентов.

## ДОПОЛНИТЕЛЬНАЯ ИНФОРМАЦИЯ

Источники финансирования. Грант Министерства науки и высшего образования РФ. Соглашение 075-15-2022-310 от 20.04.2022.

Конфликт интересов. Авторы декларируют отсутствие явных и потенциальных конфликтов интересов, связанных с содержанием настоящей статьи.

Участие авторов. Пылина С.В. — сбор и обработка материала, анализ литературных данных, написание основного текста статьи; Горбачева А.М. — сбор и обработка материала, анализ литературных данных; Елфимова А.Р. — статистический анализ данных, дизайн исследования; Еремкина А.К., Мокрышева Н.Г. — концепция и дизайн исследования, внесение в рукопись существенных правок.
